# PacBio full-length transcriptome and WGCNA reveal varietal co-expression networks and regulatory dynamics during tea plant (*Camellia sinensis*) bud dormancy

**DOI:** 10.3389/fpls.2026.1738399

**Published:** 2026-04-27

**Authors:** Xiuming Zhai, Jie Li, Fuliang Xiao, Min Tang, Yujia Hou, Surong Gao, Wei Zhang

**Affiliations:** 1 Chongqing Academy of Agricultural Sciences, Chongqing, China; 2 Agricultural Technology Extension Center of Pengshui Miao and Tujia Autonomous County, Chongqing, China; 3 Economic Crop Technology Extension Station of Yongchuan District, Chongqing, China

**Keywords:** alternative splicing, bud dormancy, *Camellia sinensis*, full-length transcriptome, WGCNA

## Abstract

Bud dormancy in tea tree (*Camellia sinensis*) is a key adaptive trait that strongly influences tea yield and quality. However, its molecular basis remains less well characterized than that of model plants and deciduous fruit trees. Previous studies have linked hormone homeostasis, carbohydrate status, and stress signaling to dormancy regulation, but most have relied on short-read RNA-seq or candidate-gene approaches, leaving isoform diversity and network-level regulation insufficiently resolved. Here, we combined PacBio Iso-Seq with expression profiling and weighted gene co-expression network analysis (WGCNA) to investigate three tea cultivars with contrasting dormancy characteristics: YC4H (short dormancy), FD (early bud break), and YH3/YC3H (intermediate dormancy). PacBio sequencing generated high-quality full-length transcripts and enabled accurate characterization of transcript structures without assembly. This dataset revealed 151,674-186,949 cultivar-specific transcripts, extensive alternative splicing events (including 7,130 retained-intron events in FD_Mix), and 93,072-100,010 candidate long non-coding RNAs (lncRNAs), including a high-confidence subset supported by all four prediction tools. PacBio-derived transcripts were then used as a reference for Illumina-based expression quantification, which supported transcript-level analyses and WGCNA. Global expression profiling and principal component analysis showed clear separation among cultivars and dormancy stages. Differential expression and co-expression analyses identified dynamic transcriptomic changes associated with phytohormone signaling, carbohydrate metabolism, stress responses, cell-cycle reactivation, and protein quality control. WGCNA identified 20 co-expression modules showing cultivar- and stage-associated patterns. Together, these results provide a transcriptome resource and a set of candidate regulatory pathways and hub genes associated with tea bud dormancy transitions, and they offer a useful framework for future functional studies and breeding-oriented investigations.

## 1 Introduction

Tea (*Camellia sinensis* (L.) O. Kuntze) is one of the most important perennial evergreen cash crops worldwide and is the source plant for one of the most widely consumed beverages globally (Zakir et al., 2023). As a thermophilic perennial, tea exhibits rhythmic growth patterns that are strongly influenced by environmental cues, especially photoperiod and temperature (Hao et al., 2017). Bud dormancy is therefore an important adaptive mechanism that helps synchronize growth with seasonal conditions (Zhao et al., 2025b; Cooke et al., 2012).

In tea plant, two dormancy-related states have been described: banjhi dormancy and winter dormancy (Vyas and Kumar, 2005; Thirugnanasambantham et al., 2013). Winter dormancy occurs during the cold season and is mainly regulated by photoperiod and temperature, whereas banjhi dormancy is a temporary growth arrest that can occur during the growing season (Maurya and Bhalerao, 2017). The duration and depth of bud dormancy influence bud break timing, subsequent shoot growth, and ultimately tea yield and quality (Krishnaraj et al., 2011). Tea bud dormancy is characterized by suppressed growth, reduced metabolic activity, arrested cell division, and enhanced stress tolerance. During winter dormancy, buds remain quiescent until sufficient chilling has accumulated and temperatures rise to permit renewed growth. The depth and duration of dormancy vary substantially among cultivars, making dormancy regulation an important trait for tea production and climate adaptation.

The regulation of bud dormancy in perennial plants involves complex interactions between environmental signals and endogenous molecular networks (Sabir et al., 2025; Chiatante et al., 2021). Numerous studies have shown that several phytohormones, including abscisic acid (ABA), gibberellins (GAs), auxin, and cytokinins, participate in dormancy establishment and release (Parwez et al., 2022; Bano et al., 2023; Chen et al., 2020). In general, ABA is associated with dormancy induction and maintenance, whereas GAs are frequently associated with dormancy release and growth recovery (Cantoro et al., 2013; Veerabagu et al., 2023).

At the molecular level, dormancy regulation involves transcription factors, epigenetic regulation, and post-transcriptional control (Luján-Soto and Dinkova, 2021; Wang et al., 2025). Key gene families implicated in dormancy control include dormancy-associated MADS-box (DAM) genes, PHYTOCHROME genes, FLOWERING LOCUS T (FT), and CONSTANS (CO) (Niu et al., 2016; Quesada-Traver et al., 2022). Recent studies have also highlighted possible roles for alternative splicing, long non-coding RNAs (lncRNAs), and chromatin remodeling during dormancy regulation (Motor et al., 2025; Sacharowski et al., 2025; Calixto et al., 2018; Filichkin et al., 2010; Wang et al., 2014).

Despite progress in model plants and several tree species, the molecular basis of bud dormancy in tea remains comparatively understudied. The complexity of dormancy regulation, together with the perennial evergreen growth habit of tea, requires a more comprehensive transcriptome-level framework to resolve cultivar-specific and stage-specific regulatory programs.

Conventional short-read RNA-seq (RNA-seq) methods, while powerful in gene expression analysis, have limitations in accurately characterizing transcriptome complexity, particularly with respect to alternative splicing events, de novo transcript discovery, and precise gene structure annotation (Haas et al., 2013; Filichkin et al., 2010). Short-read RNA-seq cannot reliably resolve full-length transcript isoforms, often misassembles alternatively spliced variants, and struggles with accurate quantification of transcript-level expression, especially for genes with multiple isoforms. These limitations are particularly evident in non-model species with complex genomes and extensive alternative splicing patterns (Ringeling et al., 2022). For tea plants, which exhibit extensive alternative splicing as revealed in our study, these limitations could significantly impede the identification of isoform-specific regulatory mechanisms that are critical for understanding dormancy transitions.

PacBio single-molecule real-time (SMRT) sequencing addresses many of these limitations by generating full-length transcript sequences without transcript assembly. This technology is well suited for identifying alternative splicing events, discovering novel transcripts, and improving lncRNA characterization (Zhou et al., 2022; Mou et al., 2023). Its long-read capability is particularly useful for dissecting complex regulatory networks and transcriptome diversity (Yang et al., 2021).

Weighted gene co-expression network analysis (WGCNA) is a systems-level approach for identifying co-expressed modules and their associations with phenotypic traits or experimental conditions (Langfelder and Horvath, 2008). WGCNA has been widely used to study plant development, stress responses, and metabolic regulation (Pei et al., 2017). When combined with comprehensive transcriptomic data, it can help prioritize hub genes and regulatory modules associated with complex biological processes (Dileo et al., 2011; Zhang and Wong, 2022).

The main objective of this study was to characterize the transcriptomic features associated with tea bud dormancy using PacBio full-length transcriptome sequencing integrated with expression profiling and WGCNA. Specifically, we aimed to: (1) generate high-quality full-length transcriptomes for three tea cultivars (YC4H, FD, and YH3/YC3H) with contrasting dormancy characteristics; (2) characterize transcript structures, alternative splicing events, lncRNAs, and transcription factor families; (3) identify genes and transcripts showing stage- or cultivar-associated expression patterns during dormancy transitions; and (4) construct co-expression networks to identify modules and hub genes associated with dormancy-related variation. Our goal was to provide a transcriptome resource and a candidate regulatory framework for future functional and breeding studies in tea.

## 2 Results

### 2.1 PacBio full-length transcriptome assembly and quality assessment

**Figure 1:**
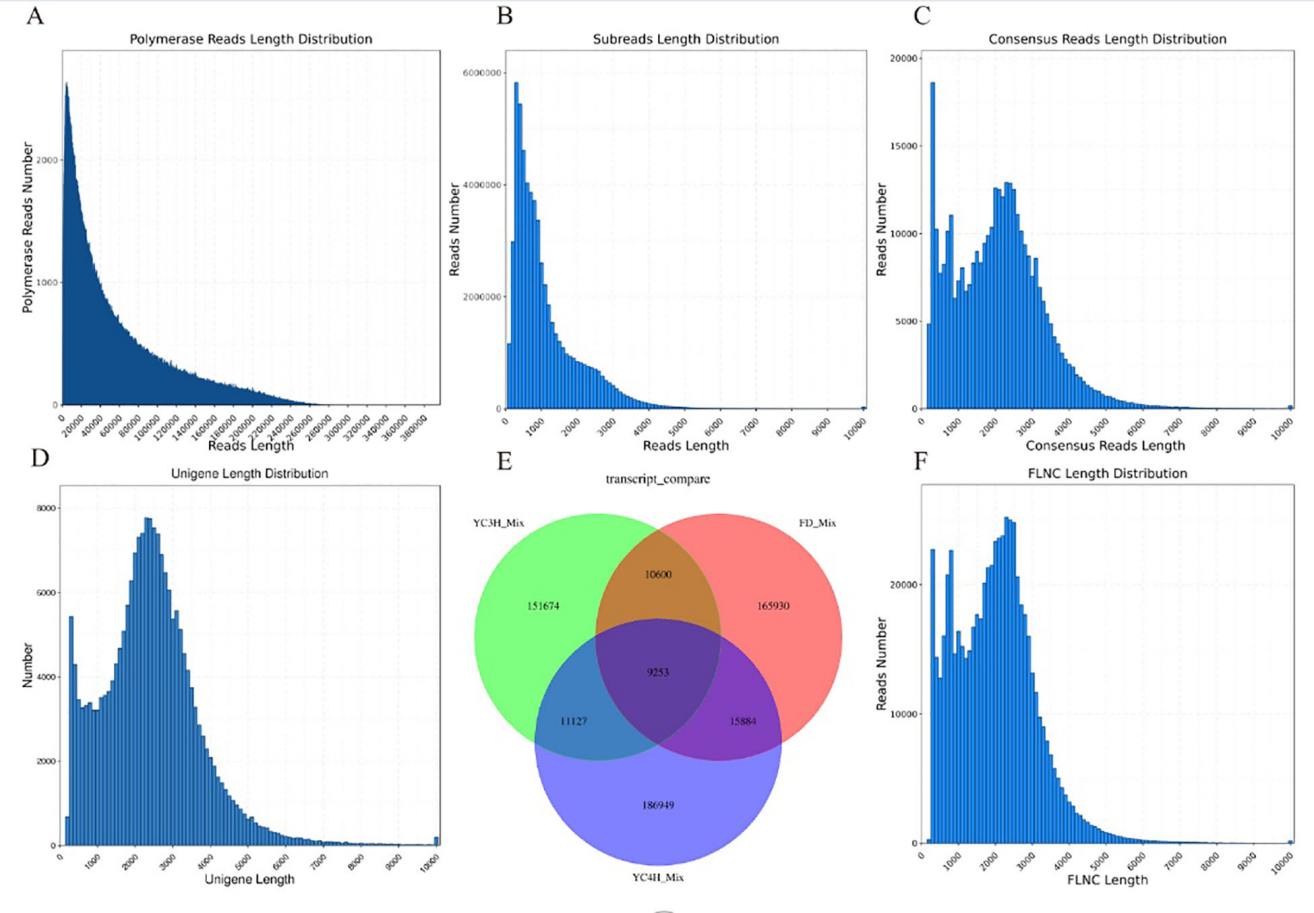
PacBio full-length transcriptome assembly and quality assessment. **(A)** Distribution of subread lengths. **(B)** Total number of subreads generated. **(C)** Length distribution of consensus sequences. **(D)** Length distribution of assembled unigenes. **(E)** Venn diagram of shared and unique transcripts among three tea varieties. **(F)** Distribution of full-length non-chimeric (FLNC) read lengths.

To investigate the molecular features associated with bud dormancy in tea, we performed PacBio Iso-Seq sequencing on three cultivars with contrasting dormancy phenotypes: YC4H (short dormancy), FD (early bud break), and YH3/YC3H (intermediate dormancy). After filtering and transcript assembly, high-quality full-length transcriptome datasets were obtained for all three cultivars (**Figure 1**).

Sequencing generated large, high-quality datasets for all three cultivars. YC3H_Mix produced 65.5 Gb from 51,037,066 subreads, with an average length of 1,284 bp and an N50 of 2,053 bp. YC4H_Mix produced 57.54 Gb from 49,236,569 subreads, with an average length of 1,169 bp and an N50 of 2,074 bp. FD_Mix produced 64.38 Gb from 59,899,870 subreads, with an average length of 1,075 bp and an N50 of 1,702 bp (**Figures 1A, B**).

The length distribution of the consensus sequences showed a multi-peak pattern with significant peaks around 500 bp and broader peaks between 2,000 - 3,000 bp, confirming the successful generation of long and high-fidelity sequences (**Figure 1C**). The length distribution of the assembled single genes showed a bimodal pattern, successfully reconstructing many full-length transcripts (**Figure 1D**). The full-length non-chimeric (FLNC) read length distribution further validated the successful capture of many full-length transcripts up to 10,000 bp in length (**Figure 1F**).

To assess the diversity of transcriptomes among the different varieties, comparative analysis revealed many overlapping and unique transcripts among the three tea tree varieties (**Figure 1E**). A total of 9,253 transcripts among all varieties represented a core collection of conserved expressed genes. Many variety-specific transcripts were identified: 151,674 unique to YC3H_Mix, 165,930 unique to FD_Mix, and 186,949 unique to YC4H_Mix. The two-by-two overlap analysis showed many shared transcripts, 15,884 between FD_Mix and YC4H_Mix, 11,127 between YC3H_Mix and YC4H_Mix, and 10,600 between YC3H_Mix and FD_Mix.

Assembly metrics indicated that the quality of full-length transcriptome reconstruction was high for all varieties. The N50 values were stable at over 1,700 bp and the average read length was over 1,000 bp, which indicated high sequencing depth and quality. The high number of variety-specific transcripts highlights the genetic diversity among the studied varieties and their potential for different dormancy responses. These results provide a high-quality basis for downstream analysis of gene expression dynamics and identification of regulatory elements in tea tree bud dormancy.

### 2.2 Functional annotation and transcriptome characterization

**Figure 2:**
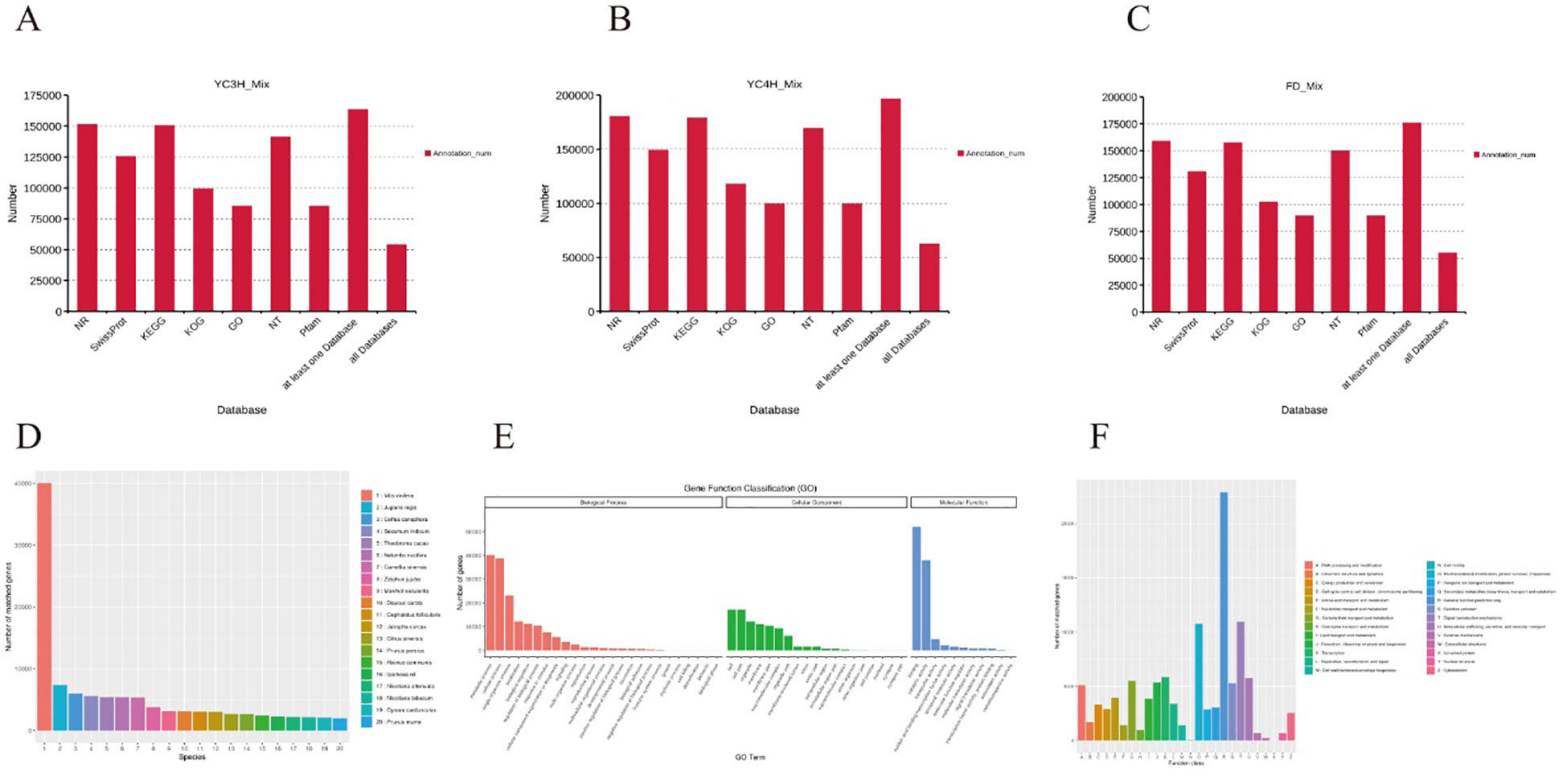
Functional annotation and transcriptome characterization. **(A–C)** Annotation statistics in different databases for YC3H_Mix, YC4H_Mix, and FD_Mix. **(D)** Top 20 species distribution of homologous genes. **(E)** Gene Ontology (GO) classification of full-length transcripts. **(F)** KEGG pathway enrichment analysis of full-length transcripts.

To assign biological functions to the assembled full-length transcripts, we annotated them against multiple public databases, including NR, SwissProt, KEGG, KOG, GO, NT, and Pfam. This multi-database strategy improved annotation coverage and provided a broad functional overview of the tea transcriptomes (**Figure 2**).

Annotation coverage was high in all three cultivars (**Figures 2A–C**). In YC3H_Mix, approximately 165,000 transcripts were annotated in at least one database, with the highest coverage in NR, SwissProt, and KEGG. YC4H_Mix showed approximately 190,000 annotated transcripts, with particularly broad coverage in NR, KEGG, and NT. FD_Mix showed a comparable pattern, with approximately 178,000 transcripts annotated in at least one database. Notably, approximately 55,000-60,000 transcripts in each cultivar were annotated across all queried databases, representing a conserved and well-supported set of transcripts.

Species distribution of annotated transcripts: BLASTX annotation against the SwissProt and NR databases revealed the taxonomic distribution of homologous sequences. The top species with highest sequence similarity to tea tree transcripts were: (1) Vitis vinifera (grape, ~44,000 matching transcripts), (2) Juglans regia (walnut, ~9,000), (3) Coffea canephora (coffee, ~7,500), (4) *Camellia sinensis* (tea tree itself, ~6,800), (5) Sesamum indicum (sesame, ~6,500), and (6) Theobroma cacao (cacao, ~6,300). The high similarity to V. vinifera and other woody perennial species is consistent with evolutionary relationships, as these species share similar genomic characteristics and gene family expansions. The presence of *C. sinensis* sequences in the database reflects previously annotated tea tree genomes. The distribution pattern reflects both evolutionary relationships and the availability of well-annotated genomes in public databases. The homology of the remaining species decreases progressively, from Chinese date or jujube (*Ziziphus jujuba*) to plum (Prunus mume), with several matching genes of about 4,500 - 2,500. This distribution pattern suggests a high degree of conservatism between tea trees and woody perennials and highlights the evolutionary relationships within the plant kingdom.

Gene Ontology analysis focusing on specific functional terms: To provide biologically meaningful functional insights, GO classification was performed with emphasis on the most specific (tip-level) terms rather than broad root terms. The complete GO annotation results are provided in **Supplementary File S1**. Here, we present the most informative specific GO terms that reflect the biological characteristics of tea bud dormancy transcriptomes. Biological processes: The most abundant specific terms (tip-level) included ‘response to hormone’ (~3,200 transcripts), ‘carbohydrate metabolic process’ (~2,800), ‘response to stress’ (~2,600), ‘cell cycle process’ (~1,900), ‘protein folding’ (~1,700), ‘DNA replication’ (~1,400), ‘oxidative stress response’ (~1,200), and ‘signal transduction’ (~1,100). These terms directly reflect key processes involved in dormancy regulation. Cellular components: Specific terms with highest representation included ‘nucleus’ (~8,500), ‘plasma membrane’ (~6,200), ‘chloroplast’ (~5,800), ‘mitochondrion’ (~4,500), ‘endoplasmic reticulum’ (~3,200), ‘ribosome’ (~2,800), and ‘cell wall’ (~2,400). Molecular functions: The most informative specific terms included ‘ATP binding’ (~5,200), ‘DNA binding’ (~4,800), ‘protein binding’ (~4,500), ‘hydrolase activity’ (~3,600), ‘kinase activity’ (~2,800), ‘transcription factor activity’ (~2,200), and ‘oxidoreductase activity’ (~2,000). This analysis reveals that the transcriptome is enriched for processes related to hormone signaling, stress responses, cell cycle regulation, and carbohydrate metabolism, which are central to dormancy regulation.

KOG classification further summarized transcript functions across 25 categories (**Figure 2F**). The most represented categories included translation, ribosome structure and biogenesis; general function prediction; post-translational modification, protein turnover and chaperones; signal transduction mechanisms; carbohydrate transport and metabolism; transcription; replication, recombination and repair; RNA processing and modification; energy production and conversion; and amino acid transport and metabolism. In contrast, categories such as cell motility, extracellular structures, and nuclear structure were comparatively less represented, indicating that these functions contribute a smaller fraction of the annotated transcriptome.

The comprehensive annotation results indicate a high-quality transcriptome assembly with broad functional coverage across multiple databases. Significant overlap between databases (55,000 - 60,000 transcripts annotated in all databases) suggests reliable functional annotation, while high coverage in at least one database (165,000 - 190,000 transcripts) ensures comprehensive characterization of functions. The high degree of homology with grape (Vitis vinifera) and other well-annotated plant species provides confidence in functional predictions and supports the conduct of comparative genomics analyses. These annotation results provide a solid foundation for understanding the molecular mechanisms of bud dormancy in tea tree and provide a valuable resource for downstream functional analyses.

### 2.3 Regulatory elements: transcription factors and long non-coding RNAs

To characterize regulatory components potentially associated with tea bud dormancy, we analyzed transcription factors (TFs) and long non-coding RNAs (lncRNAs) in the three cultivars. These analyses were intended to describe the regulatory landscape represented in the full-length transcriptomes and to highlight candidate components for downstream interpretation.

**Figure 3:**
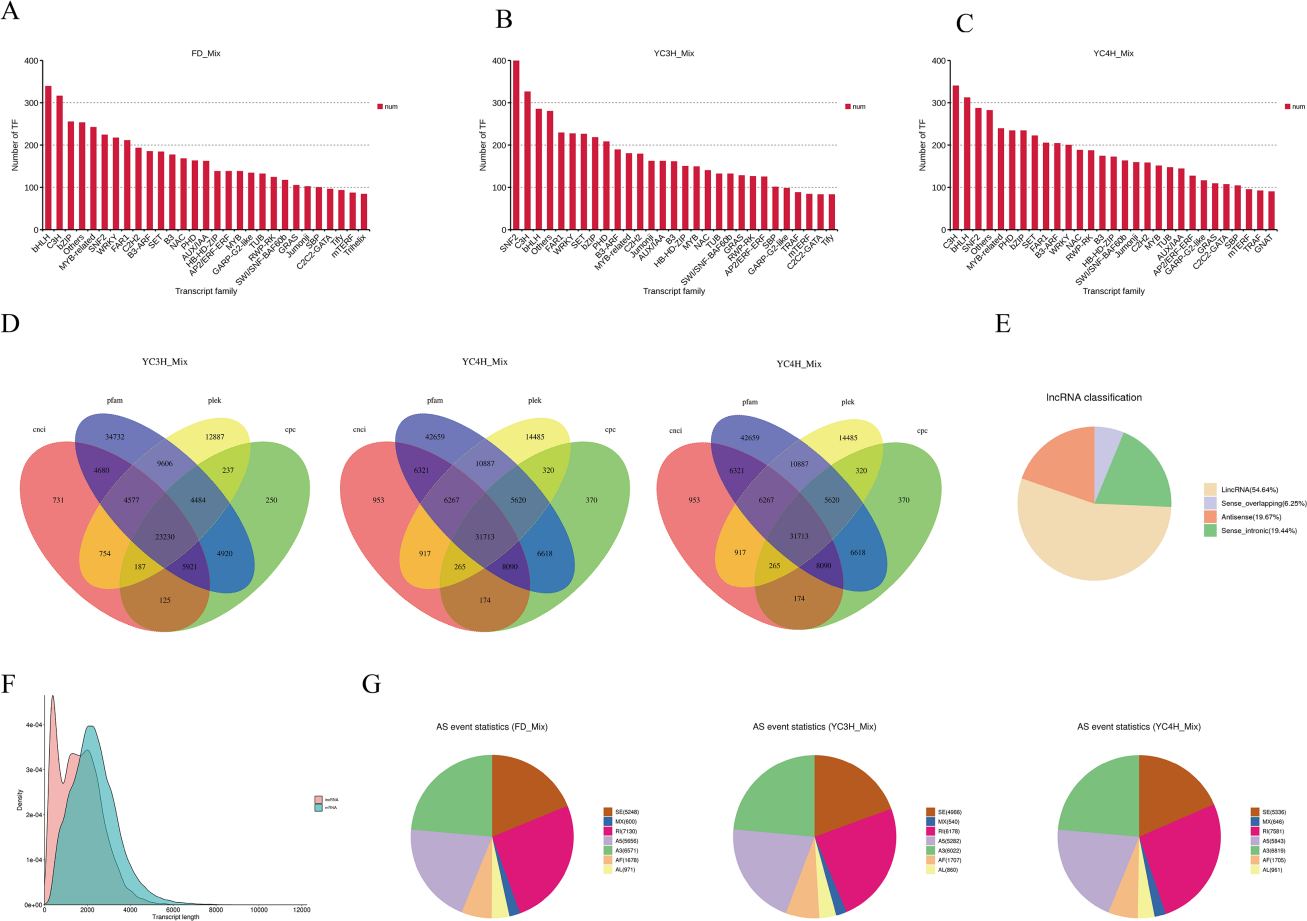
Regulatory elements: transcription factors and long non-coding RNAs. **(A–C)** Distribution of transcription factor families across FD_Mix, YC3H_Mix, and YC4H_Mix. **(D)** Overlap of transcripts/genes across defined categories. **(E)** Classification of identified long non-coding RNAs (lncRNAs). **(F)** Transcript length distribution. **(G)** Alternative splicing event statistics across tea varieties.

Many transcription factors were identified across the three transcriptomes (**Figures 3A–C**). Major TF families included C3H, MYB-related, bHLH, WRKY, bZIP, RWP-RK, SET, SNF2, FAR1, C2H2, AP2/ERF-ERF, and NAC. The prominence of MYB-related, bHLH, NAC, WRKY, and bZIP families is notable because these TF groups are widely implicated in plant development, stress responses, and hormone-associated processes. Here, they are reported as abundant TF families present in the tea dormancy transcriptomes.

To identify candidate long non-coding RNAs (lncRNAs), we applied four computational tools (CNCI, Pfam, PLEK, and CPC) (**Figure 3D**). This combined strategy identified 93,072 candidate lncRNAs in YC3H_Mix and 100,010 candidate lncRNAs in FD_Mix, while also defining a more stringent high-confidence subset supported by all four methods. In YC3H_Mix, 23,230 lncRNAs were predicted by all four tools; in FD_Mix, the corresponding high-confidence subset contained 33,169 lncRNAs. Reporting both total candidate sets and the four-tool overlap subset helps distinguish broad prediction output from more conservative confidence estimates.

The predicted lncRNAs were further classified according to their genomic positions relative to protein-coding genes (**Figure 3E**). Long intergenic non-coding RNAs (lincRNAs) were the largest category (54.64%), followed by antisense lncRNAs (19.67%), intronic lncRNAs (19.44%), and sense-overlapping lncRNAs (6.25%). This distribution indicates that intergenic transcripts constitute the major predicted lncRNA class in these tea transcriptomes, whereas antisense and intronic lncRNAs represent additional candidate regulatory components.

Length-distribution analysis showed clear differences between predicted lncRNAs and mRNAs (**Figure 3F**). Predicted lncRNAs displayed a sharper distribution toward shorter transcript lengths and declined rapidly beyond approximately 4,000 bp, whereas mRNAs showed a broader distribution extending to approximately 6,000-7,000 bp. These trends are consistent with the generally shorter transcript structures of lncRNAs relative to mRNAs.

Alternative splicing analysis revealed extensive post-transcriptional complexity across cultivars (**Figure 3G**). In FD_Mix, retained intron (RI) events were the most abundant (7,130), followed by alternative 3’ splice-site events (A3; 6,571), alternative 5’ splice-site events (A5; 5,656), and skipped exon events (SE; 5,248). Less frequent classes included alternative first exon (AF; 1,678), alternative last exon (AL; 971), and mutually exclusive exon (MX; 600) events. These results indicate that intron retention and splice-site variation are major forms of alternative splicing in the tea dormancy transcriptomes.

Together, the transcription factor, lncRNA, and alternative-splicing analyses indicate that tea bud dormancy is associated with multiple regulatory layers. In particular, the abundance of MYB-related, bHLH, NAC, WRKY, and bZIP transcription factor families, the presence of large candidate lncRNA sets, and the prevalence of retained-intron and alternative splice-site events highlight the transcriptomic complexity of dormancy transitions in tea.

### 2.4 Differential expression analysis and gene expression dynamics

**Figure 4:**
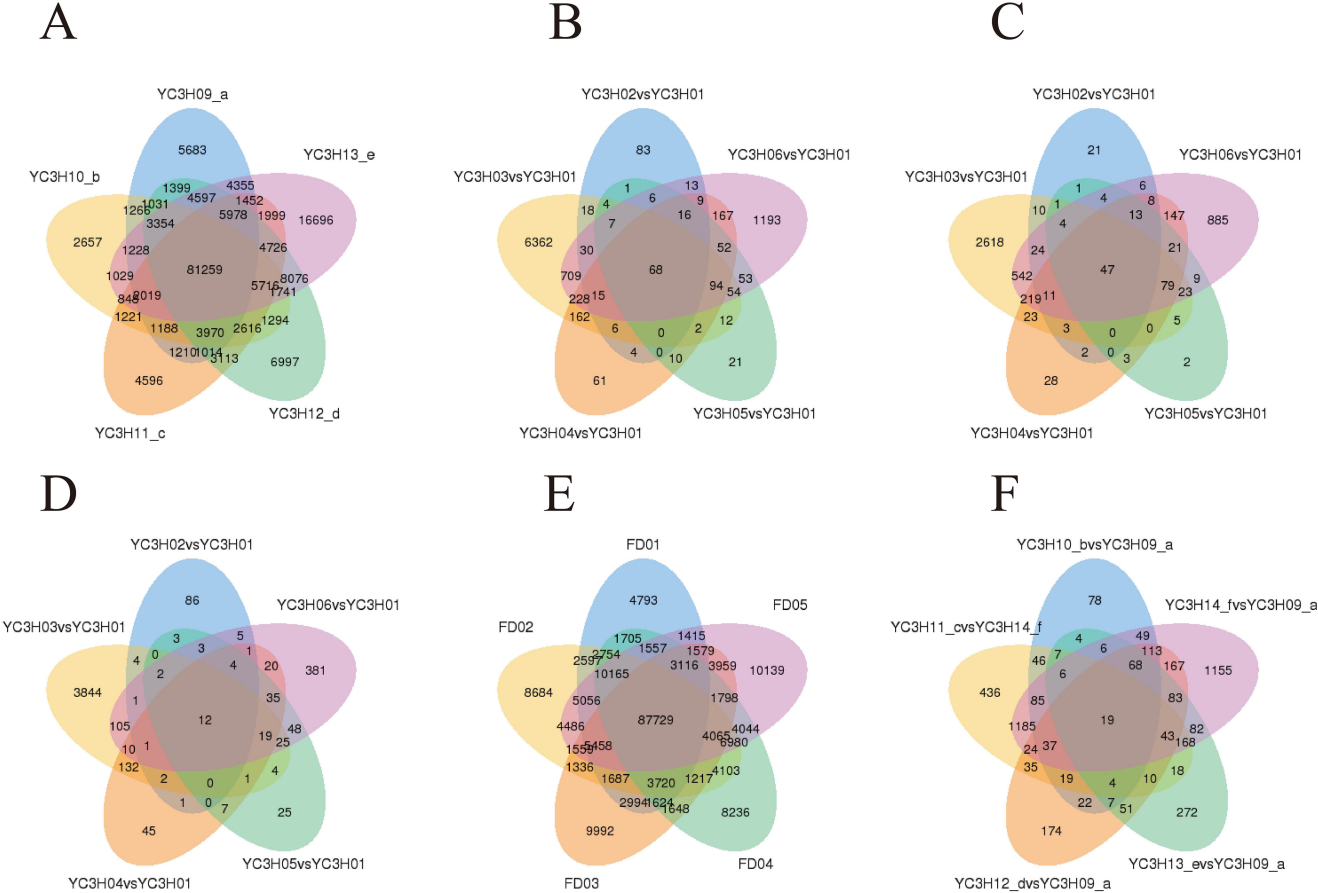
Differential expression analysis and gene expression dynamics. **(A)** Venn diagram of identified full-length transcripts across different tea plant samples. **(B)** Venn diagram of differentially expressed transcripts (DETs) in various tea plant samples compared to YC3H01. **(C)** Venn diagram of a subset of DETs. **(D)** Venn diagram of a distinct subset of DETs. **(E)** Venn diagram of identified full-length transcripts across different functional domains within the FD variety. **(F)** Venn diagram of DETs from specific pairwise comparisons in tea plant.

To identify genes and transcripts associated with dormancy transitions, we analyzed expression changes across dormancy stages and among the three tea cultivars (**Figure 4**). This analysis revealed extensive transcriptional reprogramming associated with YC3H, YC4H, and FD dormancy phenotypes.

To identify genes associated with dormancy transitions, we performed two complementary sets of comparisons: (1) intra-cultivar temporal comparisons, which tracked expression changes across sequential dormancy stages within each cultivar, and (2) inter-cultivar comparisons at approximately matched developmental stages, which were used to highlight cultivar-associated differences. Because each variety × time-point combination was represented by one pooled library, these comparisons were interpreted as a screening framework for candidate genes, transcripts, and pathways associated with dormancy progression rather than as population-level inference.

The temporal dynamics of gene expression indicate substantial transcriptome reprogramming during dormancy progression and release. Genes associated with dormancy maintenance generally tended to decrease during dormancy release, whereas genes associated with growth and metabolism tended to increase. Cultivar-specific differences were also evident: YC4H showed a relatively compressed transition during dormancy release, whereas FD displayed earlier induction of growth-associated transcripts than YH3/YC3H. These stage- and cultivar-associated patterns provide candidate molecular signatures for natural variation in tea bud dormancy.

### 2.5 Functional enrichment analysis of differentially expressed genes

**Figure 5:**
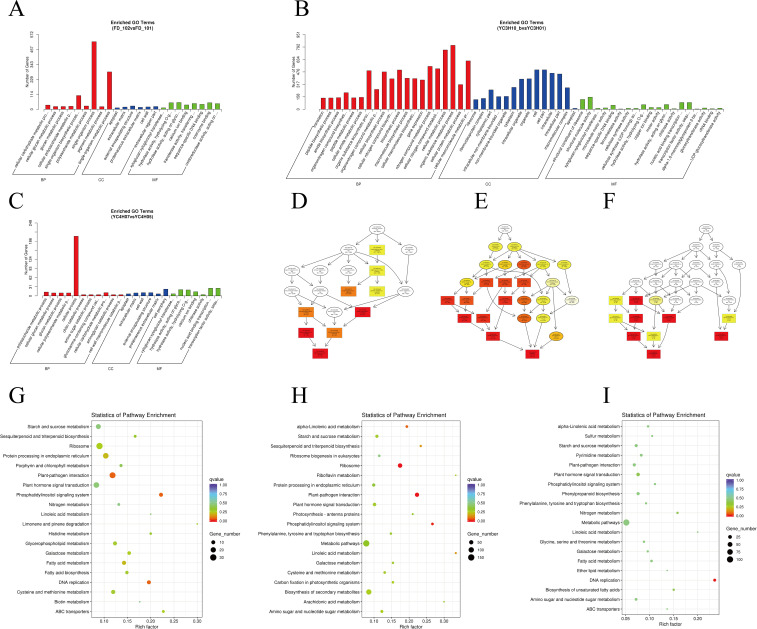
Functional enrichment analysis of differentially expressed genes. **(A)** GO terms of DEGs between FD_102 and FD_101. **(B)** GO terms of DEGs between YC3H10 and YC3H01. **(C)** GO terms of DEGs between YC3H and FD_101. **(D)** KEGG pathway analysis of DEGs between FD_102 and FD_101. **(E)** KEGG pathway analysis of DEGs between YC3H10 and YC3H01. **(F)** KEGG pathway analysis of DEGs between YC3H and FD_101. **(G)** Bubble plot of KEGG pathway enrichment of DEGs between FD_102 and FD_101. **(H)** Bubble plot of KEGG pathway enrichment of DEGs between YC3H10 and YC3H01. **(I)** Bubble plot of KEGG pathway enrichment of DEGs between YC3H and FD_101.

To evaluate the biological context of the identified differentially expressed genes (DEGs), we performed GO and KEGG enrichment analyses (**Figure 5**). These analyses were used to summarize the major biological processes and pathways represented in dormancy-associated expression changes.

GO enrichment analysis categorized DEGs into biological process, molecular function, and cellular component terms (**Figures 5A–C**). Enriched biological processes included response to stimulus, metabolic process, cellular process, and developmental process, indicating broad physiological reprogramming during dormancy transitions. In the molecular function category, catalytic activity, binding, and transporter activity were prominent, suggesting extensive enzymatic and regulatory involvement. In the cellular component category, terms related to cell wall, membrane, and organelles indicated substantial structural and subcellular remodeling.

KEGG pathway enrichment analysis identified several pathways enriched among DEGs (**Figures 5D–I**). These included plant-pathogen interaction, phosphatidylinositol signaling system, phytohormone signaling, DNA replication, protein processing in the endoplasmic reticulum, amino sugar and nucleotide sugar metabolism, and ubiquitin-mediated proteolysis. Together, these pathways suggest coordinated changes in signaling, cellular remodeling, and metabolism during dormancy progression and release.

### 2.6 Weighted gene co-expression network analysis

**Figure 6:**
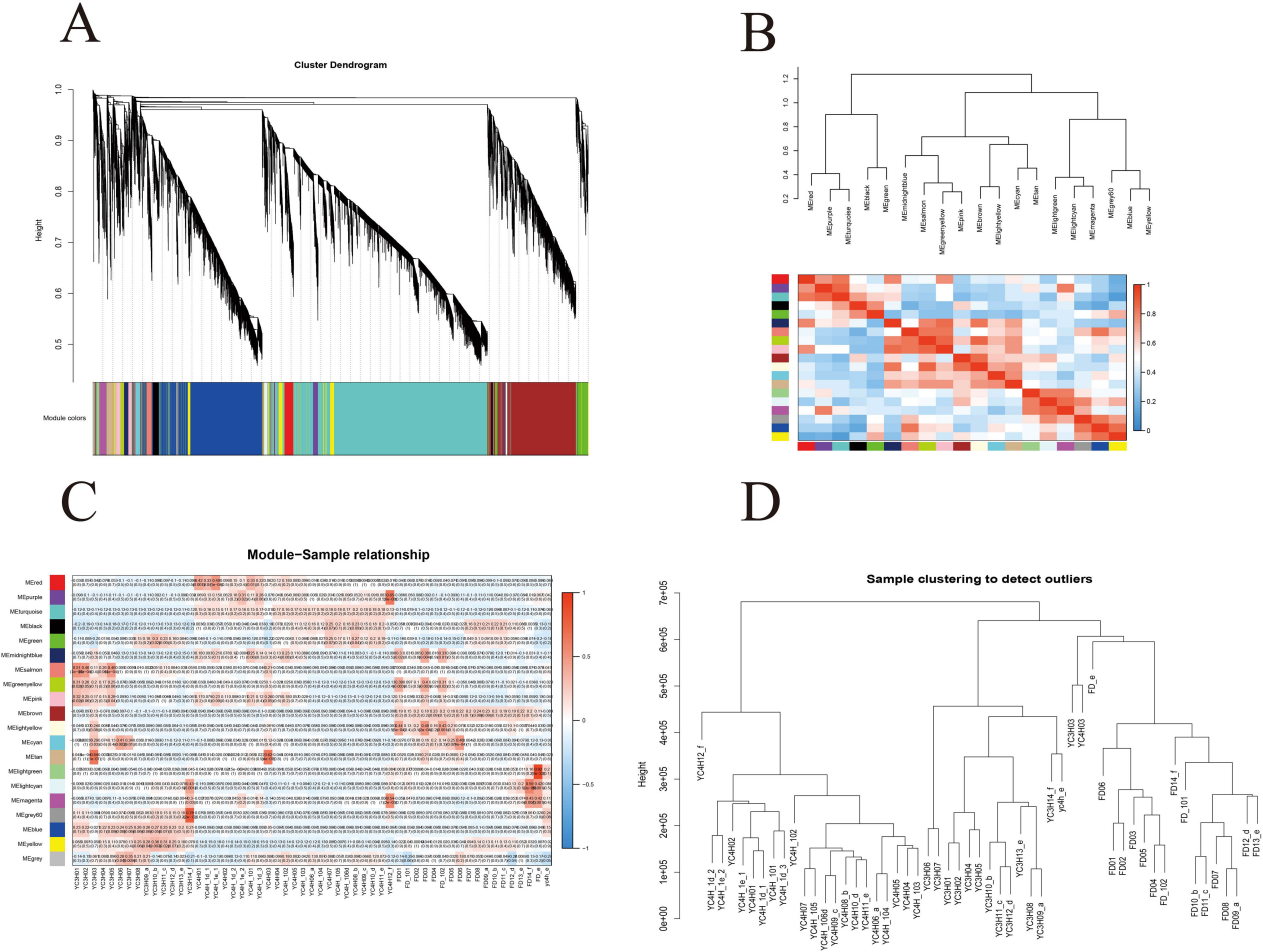
Weighted gene co-expression network analysis (WGCNA). **(A)** Clustering dendrogram of samples based on their Euclidean distance. **(B)** Hierarchical cluster tree showing co-expression modules identified by WGCNA and heat map analysis of the samples with different modules. **(C)** Module–trait association; each row corresponds to a module, and each column represents a specific variety or dormancy stage. The color of each cell at the row–column intersection indicates the correlation coefficient between a module and the traits. **(D)** Sample clustering dendrogram used to detect outliers among the analyzed libraries.

To systematically identify co-expressed gene modules associated with dormancy traits and cultivar differences, we performed WGCNA using transcript abundance values quantified from Illumina RNA-seq data against the PacBio-derived full-length transcript reference. This analysis was used to identify stage- and cultivar-associated co-expression patterns and to prioritize candidate hub genes (**Figure 6**).

WGCNA identified 20 co-expression modules based on transcript expression patterns. The sample dendrogram (**Figure 6A**) showed clustering by cultivar, with YC4H, YC3H, and FD samples forming distinct groups. One sample (YC4H12_f) appeared as a clear outlier, and FD_e showed a comparatively weaker separation within the FD cluster.

The gene clustering dendrogram (**Figure 6B**) showed distinct module groupings, including a cluster containing MEred, MEpurple, and MEturquoise, another containing MEsalmon, MEgreenyellow, MEpink, MEbrown, and MElightyellow, and a larger cluster containing MElightgreen, MElightcyan, MEmagenta, MEgrey60, MEblue, and MEyellow.

The inter-module correlation heatmap (**Figure 6C**) revealed both positive and negative relationships among modules. Strong positive correlations were observed among modules within the same dendrogram clusters, whereas negative correlations were evident between the MEred/MEpurple/MEturquoise cluster and the large cluster containing MElightgreen through MEyellow, indicating contrasting co-expression patterns among these module groups.

The module-sample relationship heatmap (**Figure 6D**) revealed cultivar-associated patterns across the 53 pooled libraries (15 YC3H, 24 YC4H, and 14 FD). YC3H samples generally showed positive correlations with many modules across time points. YC4H samples displayed a dynamic pattern, with early samples resembling YC3H and later samples showing weaker or partly negative correlations. FD samples tended to show the opposite pattern for many modules, consistent with their earlier bud-break phenotype.

The separation of samples by cultivar and the contrasting module-trait correlations suggest that these modules capture biologically meaningful expression programs associated with dormancy transitions. Positive correlations in YC3H and early YC4H samples are consistent with dormancy-associated expression states, whereas many FD samples showed the opposite trend. Hub genes in significantly associated modules included GA20ox, MCM5, PCNA, CUL1, SKP1, TIR1, WRKY33, PYL, and PP2C, based on the predefined MM and GS thresholds.

These module-level results identify candidate regulatory nodes associated with dormancy-related variation among tea cultivars and provide a focused set of targets for future validation.

## 3 Discussion

Tea bud dormancy is a key adaptive trait in perennial tea plants, yet its molecular basis remains less well resolved than in model species and several deciduous fruit trees. In this study, we combined PacBio full-length transcriptome sequencing with Illumina-based expression quantification and WGCNA to characterize transcriptomic patterns associated with dormancy transitions in three tea cultivars with contrasting phenology. The results identify candidate pathways, modules, and hub genes linked to cultivar- and stage-associated dormancy programs, while also providing a transcript-level resource for future mechanistic studies.

### 3.1 ABA-GA antagonism and auxin/cytokinin-associated programs are associated with dormancy transitions

ABA and GA are widely implicated in dormancy regulation across perennial plants (Zhao et al., 2022; Xie et al., 2024). In our dataset, phytohormone signaling was repeatedly enriched, and transcripts related to ABA signaling (including PYL/PYR, SnRK2, ABF/AREB, and PP2C) were relatively abundant in deeply dormant samples. These expression patterns are consistent with an ABA-associated program during dormancy maintenance, as reported in previous studies of perennial dormancy (Sabir et al., 2025; Chen et al., 2020).

During dormancy release, transcripts associated with GA biosynthesis and signaling, including GA20ox, GA3ox, GID1, and DELLA-related components, showed stage- and cultivar-associated changes, particularly in FD and YC4H. FD tended to show earlier up-regulation of GA-associated transcripts and earlier reduction of some ABA-responsive markers than YH3/YC3H. These patterns are consistent with, but do not by themselves demonstrate, a shift from ABA-associated to GA-associated regulation during bud break (Rinne et al., 2011; Zhang et al., 2023).

In addition to ABA- and GA-related genes, auxin-related components (TIR1/AFB, AUX/IAA, ARF, and PIN transporters) and cytokinin signaling genes (AHK, AHP, and ARR-B) were enriched in modules associated with dormancy release (Zhao et al., 2025a). Together, these transcript patterns support a working model in which multiple hormone-associated programs are coordinately reconfigured during the transition from dormancy maintenance to renewed growth, with earlier changes in FD and a more compressed transition in YC4H.

### 3.2 Cell cycle-related transcriptional reactivation is associated with the transition from dormancy to active growth

Re-entry into the cell cycle is a hallmark of bud break in perennial plants (Prudente and Paiva, 2018). In our dataset, pathways related to DNA replication and the cell cycle were enriched in modules associated with dormancy release. Licensing factors (MCM2-7, ORC1/2, CDC6, CDT1), S-phase markers such as PCNA, cyclins, and CDKs all showed increased expression in release-associated samples, particularly in FD (Menges et al., 2005).

SKP1 emerged as a hub gene in release-associated modules. Because SKP1 is a core component of SCF complexes, its expression pattern is of interest in the context of dormancy release and renewed growth (Liu and Sherif, 2019). In YC4H, several cell-cycle-related genes showed a relatively sharp increase near bud break, whereas YH3/YC3H showed a more gradual trajectory. These expression differences suggest that cell-cycle reactivation may proceed with different timing and intensity among cultivars.

### 3.3 Carbohydrate remobilization signatures are associated with energetic and signaling shifts during meristem activation

Pathways related to starch and sucrose metabolism and amino sugar and nucleotide sugar metabolism were repeatedly enriched in release-associated analyses. Transcripts encoding AMY/BAM, SUSY, CWINV, SPS, and HXK showed coordinated increases during dormancy release, and FD generally displayed earlier or stronger changes than the other cultivars. These data are consistent with a role for carbohydrate remobilization during renewed meristem activity.

Trehalose-pathway genes (TPS and TPP) and the sugar-signaling regulator SnRK1 were co-expressed with growth-associated genes. Together with the enrichment of carbohydrate-metabolism pathways, these patterns are consistent with sugar signaling and carbon remobilization contributing to dormancy release and early bud growth in tea (Nunes et al., 2013; Tsai and Gazzarrini, 2014).

### 3.4 Protein quality control and ubiquitin-proteasome pathways are enriched during dormancy release-associated stages

Protein processing in the endoplasmic reticulum and ubiquitin-mediated proteolysis were enriched in release-associated modules. Chaperones (BiP/HSP70, HSP90, and PDIs), endoplasmic-reticulum-associated degradation components, SCF/CUL4-related E3 ligases, and proteasome-related transcripts all increased during release-associated stages. These patterns are consistent with active proteome remodeling during the transition from dormancy to growth. CUL1 and SKP1 were identified as hub genes in YC4H release-associated modules, further supporting the relevance of ubiquitin-mediated turnover in this cultivar.

### 3.5 Stress-responsive pathways regulate dormancy threshold and environmental sensitivity

Winter bud dormancy helps perennial plants tolerate unfavorable environmental conditions, especially low temperature and dehydration. Consistent with this expectation, stress-related genes, including CBF/DREB and ERF transcription factors, LEA/dehydrin genes, and ROS-scavenging enzymes, were enriched in modules associated with stress buffering and dormancy maintenance (Ding et al., 2019; Harfouche et al., 2014). We also detected enrichment of phosphatidylinositol signaling and MAPK-related components, suggesting that environmental signal integration may be associated with dormancy-state transitions.

YC3H maintained a relatively stronger stress-associated expression profile in late winter than YC4H, whereas YC4H showed more rapid attenuation of several stress-related programs near bud break. These differences may reflect cultivar-specific strategies for balancing environmental buffering and growth reactivation. We also observed enrichment of plant-pathogen interaction components, including FLS2-, EFR-, and CDPK-related transcripts, suggesting potential overlap between stress- and defense-associated signaling during dormancy transitions (Hung et al., 2014). However, these interpretations remain hypothesis-generating and require functional validation.

### 3.6 Weighted gene co-expression network analysis identifies variety-specific co-expression programs

WGCNA is useful for identifying coordinated expression programs in complex developmental transitions (Singh et al., 2017). In the present study, the co-expression network highlighted clear cultivar-associated patterns and helped prioritize candidate modules and hub genes associated with tea bud dormancy.

YC3H samples showed broadly positive correlations with many modules across the dormancy period, whereas YC4H displayed a more dynamic shift from early positive correlations to weaker or negative correlations in later samples. These contrasting patterns suggest that the timing and coordination of transcriptome reprogramming differ among cultivars.

FD samples showed broadly opposite module-correlation patterns relative to many YC3H and early YC4H samples, and FD-associated modules were enriched for DNA replication, GA-related signaling, and carbohydrate metabolism. Hub genes in these modules included MCM5, PCNA, GA20ox, MYB3R, and SUSY. YC4H-associated modules were enriched for ubiquitin-mediated proteolysis and auxin-related signaling and included hub genes such as CUL1, SKP1, TIR1, PIN1, and NAC-family transcription factors. Together, these patterns suggest that different cultivars may deploy partially distinct regulatory programs during dormancy release.

### 3.7 Multiple levels of transcriptional and post-transcriptional regulation

Transcription factor networks are important regulators of developmental transitions in plants (Jiang et al., 2014). In our dataset, MYB-related, bHLH, NAC, WRKY, and bZIP families were prominent among identified transcription factors and among genes present in dormancy-associated modules (Puccio et al., 2022). Their distribution across modules is consistent with possible roles in growth regulation, stress responses, and hormone-associated processes, but the specific functions of these tea genes remain to be tested experimentally.

Our analyses also highlighted extensive lncRNA representation and widespread alternative splicing (Calixto et al., 2018; Filichkin et al., 2010; Wang et al., 2014). Several lncRNAs were highly connected to modules containing hormone-associated genes such as PYL, PP2C, and GID1/DELLA-related transcripts, suggesting that they may be candidate regulators of dormancy-related expression programs. Likewise, alternative splicing events affecting PP2C-, ARF-, and DELLA-related transcripts point to possible post-transcriptional modulation during dormancy transitions. These observations extend the candidate regulatory framework for tea dormancy but remain association-based and will require targeted validation.

### 3.8 Integrated regulatory framework for tea bud dormancy and implications for crop improvement

Taken together, our data support a working model in which dormancy maintenance is associated with ABA-related signaling, stress-response programs, and protective pathways, whereas dormancy release is associated with increased expression of GA-related genes, auxin/cytokinin-associated components, carbohydrate-remobilization genes, proteostasis-related pathways, and cell-cycle regulators. This model is intended as a transcriptome-based hypothesis framework rather than a causal demonstration of mechanism.

An important implication of these results is that dormancy should not be viewed as a uniform state across tea cultivars. Instead, the timing and relative strength of hormone-associated, stress-associated, metabolic, and cell-cycle-related programs appear to differ among FD, YC4H, and YH3/YC3H. This cultivar-dependent modularity may help explain natural variation in bud-break timing and dormancy depth and provides a basis for future comparative studies.

The hub genes identified here, including GA20ox, MCM5, PCNA, CUL1/SKP1/TIR1, WRKY33, PYL, and PP2C, should be regarded as candidate targets for future functional validation rather than confirmed regulators. Genetic perturbation, hormone measurements, and targeted molecular assays will be needed to test their causal roles in tea dormancy regulation. Nevertheless, these genes, together with the associated splice variants and lncRNAs, provide a practical shortlist for follow-up studies and for marker-oriented evaluation in breeding materials.

In summary, this study provides a transcriptome-level resource for investigating tea bud dormancy and highlights cultivar-associated pathways, modules, and candidate hub genes linked to dormancy transitions. By integrating full-length transcript discovery with Illumina-based expression quantification and co-expression analysis, we establish a framework for future functional studies of dormancy regulation in tea. Because the present inferences are based on transcript abundance patterns, the proposed regulatory relationships should be considered candidate models that require further validation.

## 4 Materials and methods

### 4.1 Plant materials and sampling

Three tea cultivars with contrasting dormancy characteristics were analyzed: YC4H (short dormancy), FD (early bud break), and YH3, which corresponds to the YC3H sample label used in the sequencing datasets. These cultivars were maintained under standard cultivation conditions at the Tea Research Institute of the Chinese Academy of Agricultural Sciences and were selected to capture natural variation in dormancy timing and depth.

Plants were grown under field conditions in Chongqing, China (28°N, 105°E). All sampled plants were 8–10 years old and were managed using standard agronomic practices. Dormancy phenotyping was based on bud-break percentage and growth-stage progression during the sampling period.

Sampling design and time points: Axillary buds were collected from September 2018 to April 2019 at 15-day intervals. Sampling stages were classified as early dormancy (September to mid-October), deep dormancy (mid-October to January), dormancy release (February to March), and bud break (late March to April). The dataset comprised 15 YC3H libraries, 24 YC4H libraries, and 14 FD libraries, for a total of 53 libraries. Each library represented a pooled axillary-bud sample collected from 10–15 plants of the same cultivar at a given time point to reduce individual-plant variation and to provide sufficient RNA for sequencing. Thus, one pooled library was analyzed for each cultivar × time-point combination. Samples were immediately frozen in liquid nitrogen and stored at -80 °C until RNA extraction.

### 4.2 RNA extraction, library construction and PacBio sequencing

Total RNA was extracted from frozen axillary buds using TRIzol reagent according to the manufacturer’s instructions. RNA quantity and quality were assessed using a NanoDrop 2000 spectrophotometer and a Qubit 2.0 fluorometer, and only samples with RNA integrity number (RIN) > 7.5 were used for library preparation. PacBio Iso-Seq libraries were constructed from poly(A)+ RNA using the SMARTer PCR cDNA Synthesis Kit, followed by size selection with BluePippin and SMRTbell library preparation according to PacBio protocols. PacBio sequencing was performed on the Sequel II platform. For expression quantification, Illumina RNA-seq libraries were prepared from the same 53 pooled samples and sequenced in paired-end 150-bp mode. Library construction and sequencing were carried out by Beijing Novogene Co., Ltd. (Beijing, China).

PacBio Iso-Seq library construction and sequencing: Full-length cDNA libraries were constructed using the PacBio Iso-Seq protocol. Poly(A)+ RNA was isolated from total RNA using oligo(dT) magnetic beads. Full-length cDNA was synthesized using the SMARTer PCR cDNA Synthesis Kit (Clontech, Mountain View, CA, USA) according to manufacturer’s instructions. Size selection was performed using BluePippin (Sage Science, Beverly, MA, USA) to enrich for transcripts >1 kb. SMRTbell libraries were prepared using the SMRTbell Template Prep Kit 1.0 (Pacific Biosciences, Menlo Park, CA, USA). Sequencing was performed on the PacBio Sequel II platform (Pacific Biosciences) using Sequel II Binding Kit 2.0 and Sequel II Sequencing Kit 2.0 with a movie time of 20 hours. For each variety, a pooled library was constructed from equal amounts of RNA from representative samples across the dormancy cycle. Library construction and PacBio sequencing were performed by Beijing Novogene Co., Ltd. (Beijing, China) following manufacturer’s protocols.

PacBio sequencing statistics: YC3H_Mix generated 65.5 Gb of data from 51,037,066 subreads with an average length of 1,284 bp and N50 of 2,053 bp. YC4H_Mix generated 57.54 Gb of data from 49,236,569 subreads with an average length of 1,169 bp and N50 of 2,074 bp. FD_Mix generated 64.38 Gb of data from 59,899,870 subreads with an average length of 1,075 bp and N50 of 1,702 bp.

Illumina RNA-seq library construction and sequencing: For expression quantification, Illumina sequencing libraries were constructed for all 53 individual samples. Total RNA was used to construct sequencing libraries using the NEBNext Ultra RNA Library Prep Kit for Illumina (New England Biolabs, Ipswich, MA, USA, catalog number E7530) according to manufacturer’s instructions. Poly(A)+ RNA was enriched using oligo(dT) magnetic beads. Fragmentation, first-strand cDNA synthesis, second-strand cDNA synthesis, end repair, adapter ligation, and PCR amplification were performed according to the kit protocol. Library quality was assessed using Agilent 2100 Bioanalyzer, and libraries were quantified using Qubit. Paired-end sequencing (2×150 bp) was performed on the Illumina NovaSeq 6000 platform (Illumina, San Diego, CA, USA). Sequencing was performed by Beijing Novogene Co., Ltd. (Beijing, China) following standard protocols.

Illumina sequencing depth: Each sample was sequenced to a depth of approximately 6–8 Gb (40–50 million paired-end reads per sample), providing sufficient depth for accurate expression quantification.

### 4.3 Bioinformatics analysis process

#### 4.3.1 Raw data processing, assembly and genome comparison

Raw PacBio reads were processed using SMRT Link v6.0 (Pacific Biosciences) to generate sub-reads, cyclic-coherent sequences (CCS), and full-length non-chimeric (FLNC) reads. Quality filtering was performed to remove adapter sequences and primer dimers, short reads (<50 bp) and low quality reads (accuracy <0.9). FLNC reads were clustered using the hierarchical nlog(n) algorithm implemented in SMRT Link to generate concordant sequences. Corrections were performed using the Arrow algorithm to correct sequencing errors and generate high-quality concordant sequences. Full-length transcripts were aligned to the Tea Tree* reference genome (CS1.0) using minimap2 (Li, 2018) with default parameters.

#### 4.3.2 Gene structure analysis and transcript classification

Gene structures were characterized by identifying coding sequences (CDS), untranslated regions (UTRs), and exon-intron boundaries using TransDecoder v5.5.0 (Haas et al., 2013) with default parameters. CDS predictions were performed with minimum ORF length of 100 nucleotides. Transcripts were categorized as known genes, novel isoforms, or new genes based on comparison with the reference tea tree genome (CS1.0) using minimap2 (Li, 2018) with parameters: -ax splice -uf -k14. Transcripts with alignment coverage >80% and identity >95% to annotated genes were classified as known isoforms. Transcripts aligning to known gene loci but with different exon-intron structures were classified as novel isoforms. Transcripts with no significant alignment to annotated genes (coverage <50% or identity <80%) were classified as new genes after confirming the absence of homology using BLASTN against the reference genome (E-value <1e-5).

#### 4.3.3 Alternative splicing and polyadenylation analysis

Alternative splicing events were identified and classified into seven types using rMATS (Shen et al., 2014): skipped exons (SE), mutually exclusive exons (MX), retained introns (RI), variable 5’ splice sites (A5), variable 3’ splice sites (A3), variable first exons (AF) and variable last exon (AL). Polyadenylation sites were identified based on full-length transcript sequences and analyzed for sequence motifs and positional preferences. Alternative splicing and polyadenylation analysis: Alternative splicing events were identified and classified into seven types using rMATS v4.1.2with the following parameters: readLength 150, anchorLength 1, tophatAnchor 8, minAnchorLength 8, maxAnchorLength 8, minIntronLength 50, maxIntronLength 500000, --minClusteringExons 0, --allow-clipping. AS events were filtered with FDR <0.05 and |ΔPSI| >0.1 (percent spliced in, PSI) to identify significantly differential splicing events. Polyadenylation sites were identified from full-length transcript sequences using TAPIS with default parameters, and poly(A) signal motifs (AAUAAA and variants) were identified within 50 nucleotides upstream of poly(A) sites using MEME suite.

#### 4.3.4 Functional annotation of genes and identification of regulatory elements

To ensure annotation quality while minimizing database-specific bias, we used a hierarchical annotation strategy. Transcripts were first searched against SwissProt using BLASTX (E-value < 1e-5), and unannotated transcripts were then searched against NR using the same threshold. GO terms were assigned with Blast2GO v5.2, KEGG annotations were obtained using KAAS with the bi-directional best-hit method, KOG annotations were generated using RPS-BLAST (E-value < 1e-5), and Pfam domains were identified with HMMER v3.3. Transcription factors were predicted using PlantTFDB. Candidate lncRNAs were predicted with CPC, CNCI, Pfam, and PLEK, and their genomic positions were used to classify them into lincRNA, antisense, intronic, and sense-overlapping categories.

LncRNA prediction was performed with four tools to increase confidence. CNCI v2 and PLEK v1.2 were run with default parameters to classify coding versus non-coding sequences on the basis of sequence composition. CPC v0.9-r2 was used to evaluate coding potential from ORF features and sequence similarity, and Pfam v33.1 was used to identify transcripts with known protein domains; transcripts with significant Pfam hits (E-value < 0.001) were excluded from the non-coding set.

Transcripts predicted as non-coding by at least two tools were retained as candidate lncRNAs, whereas transcripts supported by all four tools were defined as the high-confidence lncRNA subset reported in the Results. This distinction was used to avoid overinterpretation of permissive prediction output.

To determine the taxonomic distribution of homologous sequences, BLASTX searches were performed against the SwissProt and NR databases (downloaded March 2021) using BLASTX v2.11.0+ (Altschul et al., 1990) with the following parameters: E-value threshold <1e-5, maximum number of hits = 5, output format = tabular. For each transcript, the top BLAST hit (lowest E-value) was used to assign species origin. Species names were extracted from the hit description line and counted. Species distribution reflects both evolutionary relationships and the availability of well-annotated genomes in public databases.

#### 4.3.5 Fusion gene identification and expression quantification

Potential fusion transcripts were screened from full-length transcriptome data using split-alignment patterns and expression support. For expression quantification, Illumina RNA-seq data from the same 53 pooled libraries were quantified against the PacBio-derived reference transcriptome using Salmon, and transcript abundance was summarized as TPM. PacBio long reads were used to improve reference transcript structure, whereas Illumina short reads were used for expression measurement in downstream differential-expression and co-expression analyses.

#### 4.3.6 Differential expression analysis

Differentially expressed genes (DEGs) and transcripts (DETs) were screened using DESeq2 (Love et al., 2014) with |log2FoldChange| >= 1 and adjusted p < 0.05. Because each cultivar × time-point combination was represented by one pooled library, DEG/DET results were interpreted as an exploratory prioritization of candidate genes and pathways associated with dormancy transitions rather than as formal population-level inference.

#### 4.3.7 Functional enrichment analysis

Functional enrichment analysis of DEGs/DETs was performed using GO and KEGG databases. Enrichment significance was assessed using Fisher’s exact test with FDR correction.

#### 4.3.8 Weighted gene co-expression network analysis

WGCNA was performed using transcript abundance values derived from Illumina short-read RNA-seq mapped to the PacBio full-length transcript reference. PacBio reads were used to define transcript structures, including novel isoforms and lncRNAs, and Illumina data were used for quantitative expression profiling. A signed network was constructed in WGCNA v1.70–3 with soft-threshold power beta = 14, minModuleSize = 30, deepSplit = 2, and mergeCutHeight = 0.25. Module eigengenes were correlated with cultivar identity and dormancy stage, and modules with |correlation| > 0.5 and p < 0.01 were considered associated with traits. Hub genes were defined using |MM| > 0.8 and |GS| > 0.5. As with the differential-expression analyses, module-trait associations were interpreted as candidate co-expression relationships in a pooled-sample design.

## 5 Conclusions

In this study, we combined PacBio full-length transcriptome sequencing with Illumina-based expression profiling and WGCNA to investigate bud dormancy transitions in three tea cultivars with contrasting phenology. The analyses identified cultivar-associated transcriptomic changes involving phytohormone signaling, carbohydrate metabolism, stress-response pathways, proteostasis-related processes, and cell-cycle reactivation. We further prioritized candidate hub genes, including GA20ox, MCM5, PCNA, CUL1/SKP1/TIR1, WRKY33, PYL, and PP2C, together with associated splice variants and lncRNAs. These results provide a transcriptome resource and a candidate framework for future functional studies of tea bud dormancy. Because the present study is based on transcript abundance patterns from pooled libraries, the inferred regulatory relationships should be considered hypothesis-generating and require further validation.

## Data Availability

Raw sequencing data generated in this study have been deposited in the Genome Sequence Archive (GSA) at the National Ge-nomics Data Center (NGDC/CNCB) under accession number CRA037693.
